# Identification of Novel Positive Allosteric Modulators of Neurotrophin Receptors for the Treatment of Cognitive Dysfunction

**DOI:** 10.3390/cells10081871

**Published:** 2021-07-23

**Authors:** Märta Dahlström, Nather Madjid, Gunnar Nordvall, Magnus M. Halldin, Erika Vazquez-Juarez, Maria Lindskog, Johan Sandin, Bengt Winblad, Maria Eriksdotter, Pontus Forsell

**Affiliations:** 1 AlzeCure Pharma AB, 141 57 Huddinge, Sweden; Marta.Dahlstrom@alzecurepharma.com (M.D.); nather.madjid@alzecurepharma.com (N.M.); gunnar.nordvall@alzecurepharma.com (G.N.); magnus.halldin@alzecurepharma.com (M.M.H.); johan.sandin@alzecurepharma.com (J.S.); 2 Division of Clinical Geriatrics, Department of Neurobiology, Care Sciences and Society, Karolinska Institutet, 141 83 Huddinge, Sweden; maria.eriksdotter@ki.se; 3 AlzeCure Foundation, 141 57 Huddinge, Sweden; 4 Division of Neurogeriatrics, Department of Neurobiology, Care Sciences and Society, Karolinska Institutet, 171 77 Solna, Sweden; vj.erika@gmail.com (E.V.-J.); maria.lindskog@neuro.uu.se (M.L.); bengt.winblad@ki.se (B.W.); 5 Theme Inflammation and Aging, Karolinska University Hospital, 141 86 Huddinge, Sweden

**Keywords:** brain derived neurotrophic factor, nerve growth factor, TrkA, TrkB, cognition, Alzheimer’s disease

## Abstract

Alzheimer’s disease (AD) is the most common neurodegenerative disorder and results in severe neurodegeneration and progressive cognitive decline. Neurotrophins are growth factors involved in the development and survival of neurons, but also in underlying mechanisms for memory formation such as hippocampal long-term potentiation. Our aim was to identify small molecules with stimulatory effects on the signaling of two neurotrophins, the nerve growth factor (NGF) and the brain derived neurotrophic factor (BDNF). To identify molecules that could potentiate neurotrophin signaling, 25,000 molecules were screened, which led to the identification of the triazinetrione derivatives ACD855 (Ponazuril) and later on ACD856, as positive allosteric modulators of tropomyosin related kinase (Trk) receptors. ACD855 or ACD856 potentiated the cellular signaling of the neurotrophin receptors with EC_50_ values of 1.9 and 3.2 or 0.38 and 0.30 µM, respectively, for TrkA or TrkB. ACD855 increased acetylcholine levels in the hippocampus by 40% and facilitated long term potentiation in rat brain slices. The compounds acted as cognitive enhancers in a TrkB-dependent manner in several different behavioral models. Finally, the age-induced cognitive dysfunction in 18-month-old mice could be restored to the same level as found in 2-month-old mice after a single treatment of ACD856. We have identified a novel mechanism to modulate the activity of the Trk-receptors. The identification of the positive allosteric modulators of the Trk-receptors might have implications for the treatment of Alzheimer’s diseases and other diseases characterized by cognitive impairment.

## 1. Introduction

The neurotrophins are a family of growth factors involved in the development and survival of neurons. The neurotrophins, including the nerve growth factor (NGF) [1], brain-derived neurotrophic factor (BDNF) [2], and neurotrophin (NT)-3 [3,4] and NT-4/5 [5], binds the tropomyosin-related kinase (Trk) family of receptor tyrosine kinases (TrkA, TrkB and TrkC) [6]. In addition, all neurotrophins also bind to p75NTR, a member of the tumor necrosis factor receptor superfamily.

Neurotrophin signaling plays a pivotal role in hippocampal neurogenesis, synaptogenesis, and synaptic plasticity e.g., long-term potentiation [7], a cellular mechanism proposed to underlie memory formation at the level of the synapse. Several studies have shown a decrease in BDNF levels both in the hippocampus and in CSF during AD pathogenesis, suggesting that decreased BDNF signaling may contribute to the progression of hippocampal dysfunction in AD. Indeed, a growing body of experimental evidence suggests that increased BDNF signaling could enhance synaptic plasticity and improve cognition in AD [8,9,10]. The transplantation of stem cells or the lentiviral delivery of BDNF into the brains of amyloid transgenic mice or primates have shown the reversal of synapse loss and improved cognition [11]. Moreover, human carriers of the BDNF Val66Met polymorphism, which ultimately results in decreased levels of secreted BDNF, have shown increased rates of decline in memory and hippocampal atrophy relative to Val66Val carriers in the preclinical and prodromal stages of sporadic AD [12]. In line with these data, it has also been found that BDNF Val66Met polymorphism leads to impaired memory performance, decreased hippocampal glucose metabolism and increased Tau protein and phosphorylated Tau in CSF from patients with preclinical familiar AD [13]. The observed decreased secretion of BDNF in cultured hippocampal neurons transfected with the met allele suggests that reduced BDNF levels might cause decreased cognitive function [14].

Other reports also highlight the impact of the Val66Met-BDNF polymorphism on cognition with relevance in AD [15,16,17]. One study demonstrated that healthy adult BDNF Val66Met carriers with high amyloid burden showed significant and moderate to large declines in episodic memory, executive function, and language, and greater hippocampal atrophy after 36 months, compared to Val/Val homozygotes. These changes were not found in healthy adult Met carriers with a low amyloid burden [18].

Memory loss in AD has been associated with central cholinergic dysfunction in the basal forebrain (BF), from where cholinergic neurons project to the cerebral cortex and the hippocampus. Degeneration of the BF cholinergic neurons (BFCN) is one of the earliest signs of cognitive decline and precedes AD development [19,20]. Physiologically, the NGF stimulates cholinergic signaling and inflammatory pathways, but also controls amyloid precursor-protein (APP) processing, thereby reducing pathologic Aβ formation [21,22,23]. Transplantation of encapsulated cells releasing NGF to the basal forebrain in a small number of AD patients showed good safety/tolerability and supportive biomarker data, where immunohistochemical analysis and cognitive tests suggested signs of improved neuronal function and cognition in response to the secreted NGF [24,25,26].

Thus, restoration of both NGF and BDNF signaling can positively impact both BFCN and hippocampal activity, thereby potentially restoring cognitive dysfunction in AD.

Treatment with small molecules that enhance neurotrophin signaling is a mechanism that is not likely to suffer from the inherent drawbacks of neurotrophic protein per se, including costly and sometimes complicated treatments, a short half-life in plasma, local adverse events at the site of injection, and inability to pass the blood brain barrier [27]. Several attempts to identify and develop small molecule activators of neurotrophin signaling have been made, including peptidometics [28,29,30,31,32,33,34,35], small molecules with agonistic properties such as 7,8-dihydroxyflavone [36], gambogic amide [37], amitriptyline [38], LM22A [39] AIT-082 [40,41], L-783,281 and its analogues [42,43], MT-2 [44], macrocyclic compounds such as NG-011 and NG-012 [45], various natural products [46], and several both natural and synthetic substances containing a steroid backbone [47,48,49]. Interestingly, it was recently reported that different classes of anti-depressant could indeed modulate TrkB signaling, presumably by binding directly to the receptor [50].

In the present study, we describe the identification of triazinetrione derivatives, including the veterinary product Ponazuril (ACD855), as novel positive allosteric modulators of Trk-receptors, enhancing both NGF- and BDNF-signaling and acting as cognitive enhancers in multiple in vivo models.

## 2. Materials and Methods

### 2.1. Materials

The human osteosarcoma cell lines U2OS-TrkA/p75, U2OS-TrkB/p75, U2OS-TrkC/p75, U2OS-ratTrkA/p75, U2OS-FGFR1 (fibroblast growth factor receptor 1) as well as the human embryonic kidney cell line HEK293-IGF1R (insulin-like growth factor 1 receptor) from DiscoverX, Birmingham, United Kingdom, were used in the in vitro experiments. Eagle’s minimum essential medium (EMEM) ATCC #30-2003 from LGC Standards AB, Borås, Sweden; Leibovitz’s L15 medium, RPMI1640, fetal bovine serum, horse serum, penicillin/streptomycin, and Hoechst dye purchased from Invitrogen, Gothenburg, Sweden, were also used. Black poly-D-lysine coated 384-well plates (Viewplate) were purchased from PerkinElmer, Upplands Väsby, Sweden, and white 384-well assay plates(3570) were purchased from Corning, Amsterdam, Netherlands. Recombinant human NGF (450-01), BDNF (450-02), and NT3 (450-03) were obtained from PeproTech (London, UK). Recombinant intracellular domain (ICD) of TrkA (08-186) and single site biotinylated activated (08-486-20N) or non-activated TrkA (08-486-23N) were obtained from Carna Biosciences, Odense, Denmark. The chemical library used for screening was a selected subset from the libraries of the LCBKI collection (Chemical Biology Consortium, Stockholm, Sweden), Enamine, ChemDiv, Timtec, Maybridge, the NIH clinical collection, Specs, Otava, Prestwick, provided by the Karolinska High Throughput Centre (KHTC), SciLifeLab, Huddinge, Sweden, and the CBCS (Chemical Biology Consortium), Stockholm, Sweden. The selection of the 25,000 compounds to include in the screening from the available library of over 100,000 compounds used a plate-based scoring method, where plates containing many compounds with physicochemical properties suitable for CNS drugs were favored, and plates containing many compounds with poor physicochemical properties, or a few large clusters were disfavored.

### 2.2. Animal Welfare

All animal experiments and protocols were approved by the regional ethical committee at the Stockholm County court following the directives of the Swedish Animal Welfare Act 1988:534 and in accordance with directive 2010/63/EU and the Principles of Laboratory Animal Care (NIH publication No. 85-23). All efforts were made to minimize both animal suffering and the number of animals used for the study. Experiments were performed in accordance with the ethical permits N24/14, N13/15, and N138/16. All animals were allowed to habituate to the maintenance facilities and were handled by the same experimenter daily for a period of at least three days before the beginning of the experiments. All animals had free access to water and food.

### 2.3. TrkA PathHunter^®^ Cell-Based Assay and High Throughput Screening

U2OS cells were maintained subconfluent in EMEM supplemented with 10% FBS and penicillin/streptomycin/hygromycin and G418. One day prior to the experiment, 5000 cells of EMEM supplemented with 0.5% horse serum were seeded in each well of a 384-well white plate. The assay was performed as previously described [51]. Briefly, the TrkA or TrkB cell-based PathHunter^®^ assays use enzyme fragment complementation (EFC) technology for the complementation of two β-galactosidase (β-gal) fragments in order to study protein–protein interactions. Upon activation with ligand, TrkA or TrkB are phosphorylated on several tyrosine residues, including tyrosine 490, leading to an interaction with SHC-1. In the PathHunter^®^ assays, the Trk-receptors are fused to an enzyme donor tag, and SHC-1 is fused to a larger part of β-gal called an enzyme acceptor. The interactions between the recombinant enzyme donor, i.e., the Trk-receptor, with an enzyme acceptor, i.e., SHC-1, leads to an active β-gal enzyme. The functional (complemented) β-gal will hydrolyze a substrate to generate a luminescence signal. The generated luminescence was read using an EnVision plate reader from PerkinElmer, Upplands Väsby, Sweden, or an ID5 plate reader from Molecular Devices, Berkshire, RG41 5TS UK. The compound collection was screened using a single compound concentration (10 µM) at 1 or 3 ng/mL NGF. Min- and Max-control wells were included on each plate. A compound was considered a hit if the percent activation was more than 50% and if the value was two standard deviations higher than the mean value of all of the samples on the plate. The average activation of all of samples on 10 different 384-well plates was 31%. To define the dynamic range of the screening assay, we used cell culture media plus vehicle to define no activation and 30 ng/mL NGF plus vehicle to define 100% activation. After the completion of the screening, a total of 756 compounds were re-screened at two different concentrations of NGF (1 and 10 ng/mL) and at three different concentrations of compound (3, 10 and 30 µM). Due to the risk of adverse events with the systemic administration of TrkA agonists, the screen and the re-screen processes were designed to increase the likelihood of identifying the positive modulators of NGF-signaling rather than identifying TrkA agonists. Based on the results of the re-screen, in combination with the physical–chemical properties of the molecules, a collection of hits was used for the EC_50_ determinations. These molecules were tested in the presence of 0, 1, and 10 ng/mL of either NGF or BDNF with TrkA- or TrkB-expressing cells, respectively.

### 2.4. Immunoprecipitation and Kinase Activity Assay of Hemagglutinin (HA)-Tagged Full Length TrkA

A cDNA expression plasmid (pCMV3) with C-terminal-HA tagged human NTRK1 cDNA subcloned between the HindIII and XbaI sites was obtained from SinoBiological (NordicBiosite, Sweden). The human neuroblastoma cell line SH-SY5Y (ATCC, LGC Standards GmbH, Wesel, Germany) was transfected with lipofectamine 3000 and 2 µg of the plasmid. Hygromycin was used for the selection, and expansion of the TrkA-positive clones were performed using 200 µg/mL of Hygromycin. A clone with stable TrkA–HA expression was used for protein expression. Briefly, SH-SY5Y-TrkA-HA cells were seeded in T175cm2 flasks and grown until confluency. A total of 10 flasks were used for each experiment. Cells were detached using Accutase and washed twice with Dulbecco’s PBS through centrifugation. The final cell pellet was resuspended in 2 mL lysis buffer (FNN0011, Thermo Fisher Scientific, Gothenburg, Sweden) supplemented with protease and phosphatase inhibitors and 1 mM DTT. The cell suspension was sonicated with a probe sonicator at 40% amplitude for 2 × 5 s. The resulting homogenate was centrifuged for 10 min at 12,000× *g*. The pellet was discarded, and the supernatant was mixed with 100 µL anti-HA agarose/mL supernatant and incubated for 3 h on ice with gentle mixing. Thereafter, the beads were washed twice in assay buffer through centrifugation at 4000× *g*. The final immunoprecipitated TrkA-HA-beads were resuspended in 250 µL of kinase assay buffer supplemented with 100 nM of LANCE Ultra ULight™-poly GAT (PerkinElmer, Upplands Väsby, Sweden) and 1 mM DTT. Compound, dissolved in DMSO, or only DMSO was added to yield the indicated concentrations with no more than 0.1% DMSO. 12 µL aliquots of TrkA-HA beads were, after careful mixing, dispensed into a well of a low-volume 384-well plate (ProxiPlate Plus 6008280, PerkinElmer, Upplands Väsby, Sweden), and the reaction was started by the addition of 3 µL ATP at different concentrations. The plate was incubated for 60 min at 20 °C. Thereafter, 5 µL of detection reagent, consisting of 1 mM EDTA and 150 nM of an Eu-labeled anti-phosphotyrosine antibody, was added to each well. The substrate–antibody mixture was incubated for 90 min at 20 °C. TR-FRET was measured using an excitation wavelength of 340 nm and an emission wavelength of 665 nm with an iD5 plate reader (Molecular Devices, Berkshire, RG41 5TS UK). The intensity of the light at 665 nm was proportional to the level of poly-GAT phosphorylation.

### 2.5. Affinity Labeling and Immuno Precipitation/Streptavidin Adsorption

A structural analogue of ACD856 containing a primary amine, AC-0027019, was used to react with the NHS ester of Sulfo-SBED (33033, Thermo Fischer Scientific, Gothenburg, Sweden) or with NHS-Biotin (20217, Thermo Fischer Scientific, Gothenburg, Sweden) according to the manufacturer’s instructions. To ensure that most of the primary amine reacted with the NHS group, a 2-fold or 10-fold molar excess of sulfo-SBED or NHS-Biotin in relation to AC-0027019 were used for the biotinylation reaction. The reaction was conducted in 0.2 M NaHPO4 (pH8) for 60 min at 40 °C in a total volume of 125 µL. The reaction was terminated by the addition of 25 µL 2.5 M glycine. A 12,000× *g* supernatant from SHSY5Y cells expressing TrkA-HA was obtained as described above. A 0.6 mL aliquot of the supernatant was incubated with AC-0027019-biotin or AC-0027019-SBED for 30 min on ice in the dark. Thereafter, the AC-0027019-SBED containing supernatant was exposed to UV light at 365 nm at a 5 cm distance from the light source, whereas the sample containing AC-0027019-biotin was placed directly on ice. Subsequently, 100 µL of high-capacity streptavidin–agarose beads (20357, Thermo Fischer Scientific, Gothenburg, Sweden) or anti-HA agarose (A2095, Sigma Aldrich, Stockholm, Sweden) was added to the supernatant and incubated for 3 h on ice on a rotary platform. The mixture was thereafter centrifuged at 4000 rpm and washed twice with Dulbecco’s PBS. The final pellet produced by the agarose beads was dissolved in 2× loading buffer and heated for 5 min at 95 °C. 20 µL of each sample was loaded onto a Novex Bis-Tris 4–12% gel and thereafter transferred to a PVDF membrane. In essence, the last steps were similar to those described in the Western blot protocol.

### 2.6. Surface Plasmon Resonance Studies

All binding studies were performed using a Biacore T200 instrument at 25 °C (Biaffin GmbH & Co, Kassel, Germany). The analysis buffer consisted of 20 mM Tris pH 7.4, 150 mM NaCl, 50 µM EDTA, 0.5 mM TCEP, 0.05% Tween 20, and 1% DMSO. The single site biotinylated intracellular domain (amino acid residues 436–790) of TrkA (activated (08-486 20N)) or a non-activated protein (08-486 23N) was expressed as an N-terminal DYKDDDDK tagged, biotinylated protein (44 kDa) using the baculovirus expression system (Carna Biosciences, Odense, Denmark). The protein was captured on a series S Biotin CAPture sensor chip by the injection of CAPture reagent on flow cells 1–4 followed by the capturing of the activated TrkA (on flow cell 4), non-activated TrkA on flow cell 2, and the control biotinylated carbonic anhydrase protein on flow cell 3. The surface was stabilized before the compound was injected at a flow rate of 50 µL/min for interaction analyses. The surface was completely regenerated after the experiment. The integrity of the kinase domain was verified through binding studies with known TrkA-inhibitors (LOXO-101, AZ23 and GW-441756).

### 2.7. Long Term Potentiation and Electrophysiology

Male Sprague Dawley rats (4–5 weeks old) from Charles River Laboratories, Sulzfeld, Germany, were used in these experiments. Rats were deeply anesthetized using isoflurane and decapitated soon after the disappearance of the corneal reflexes. The brains were removed and placed in ice-cold standard artificial CSF (aCSF) solution (124 mM NaCl, 30 mM NaHCO_3_, 10 mM glucose, 1.25 mM NaH2PO_4_, 3.5 mM KCl, 1 mM MgCl_2_, 2 mM CaCl_2_). Hippocampal horizontal sections (400 µm thick) were prepared using a Leica VT1200S vibratome (Leica Microsystems, Kista, Sweden). Immediately after slicing, sections were transferred into an interface incubation chamber filled with standard aCSF. The chamber was held at 34 °C during the slicing period and was subsequently allowed to cool down to room temperature. After a recovery period of 2 h, slices were transferred to a submerged recording chamber with a perfusion rate of 2–3 mL/min with standard aCSF at 32 °C and bubbled with carbogen gas (5% CO_2_, 95% O_2_). The facilitation of long-term potentiation (LTP) was monitored in the Shaffer collaterals (SC)-CA1 pathway in the hippocampus. For this purpose, an extracellular recording pipette filled with regular aCSF was placed in the stratum radiatum, and field excitatory postsynaptic potentials (fEPSPs) were evoked through the electrical stimulation of the SC using a bipolar concentric electrode (FHC, Bowdoin, ME, USA). Stable fEPSP baseline responses were collected every 30 s over at least 30 min, using 50–60% of the maximal response. A subthreshold theta burst stimulation (Ø burst), used to induce a weak potentiation, was delivered through the stimulation electrode and consisted of a train with 10 bursts delivered at 5 Hz. Each burst consisted of 4 pulses at 100 Hz. Data were normalized with respect to the mean fEPSP slope recorded during the last 10 min of the baseline period.

### 2.8. In Vivo Microdialysis

The microdialysis experiments were conducted using Pronexus AB, Bromma, Sweden (ethical permit N24/14) on awake, freely moving Sprague Dawley rats (*n* = 6, 7–8 weeks old from Janvier Labs, Le Genest-Saint-Isle, France) as previously described [52,53]. On the day of the microdialysis experiment, the rat was in its home cage and placed into a frame of a single-beam locomotor activity monitoring system. Locomotor activity of rats undergoing microdialysis sampling was recorded simultaneously in 5-min intervals. A microdialysis probe (Eicom A-I: 0.22 mm O.D., 4 mm membrane length with 50 kDa cut-off) was inserted into the guide cannula of the awake rat. The probe was connected with fluorinated ethylene propylene (FEP) tubing (0.1 mm I.D.) to the balancing arm of the CMA 120 system for freely moving animals (CMA/Microdialysis) equipped with a 2-channel swivel (Microbiotech/se AB, Stockholm, Sweden). The drug solution and aCSF (vehicle) were filtered through sterile Luer syringe disc filters (0.2 μm pore size, Pall Corp., Lund, Sweden. The probe was perfused at a constant flow rate of 1 µL/min with aCSF solution (148 mM NaCl, 4 mM KCl, 0.8 mM MgCl_2_, 1.4 mM CaCl_2_, 1.2 mM Na_2_HPO_4_, 0.3 mM NaH_2_PO_4_, pH 7.2). Each rat was allowed to habituate to the new environment for 120 min. Following this stabilization period, the microdialysis samples were collected in 30-min intervals. The first 2 samples were collected to determine the basal extracellular levels of the neurotransmitters. Thereafter, the perfusion medium was switched to vehicle while collecting the samples over the following 4 h. At that point, the vehicle medium was switched to the aCSF containing ACD855 at a concentration of 30 µM, and samples were collected for an additional 4 h. Finally, the perfusion medium was switched back to aCSF, and 4 samples were collected during this wash-out period. After finalizing the experiment, the animals were sacrificed by an overdose of isoflurane and dislocation of the neck. The brains were rapidly removed, flash-frozen on dry ice, and stored at −80 °C for the additional analysis of tissue biomarkers or the histological verification of the microdialysis probe placement. Concentrations of acetylcholine (ACh) and glutamate (Glu) in the microdialysis samples were measured using ultra-high performance liquid chromatography tandem mass spectrometry (UHPLC-MS/MS), whereas the levels of the monoamines dopamine (DA), noradrenaline (NA), and 5-serotonin (5-HT) in the microdialysates were measured using ion-exchange narrow bore column liquid chromatography with electrochemical detection as described elsewhere [52,53,54].

### 2.9. Western Blot Analysis

Frozen hippocampal tissues were homogenized in cell lysis buffer supplemented with protease and phosphatase inhibitors (Cell Signaling Technology (BioNordika, Stockholm, Sweden) through sonication. The resulting homogenate was mixed with 4X sample buffer and denatured by heating the mixture at 95 °C for 2 min. Western blot was performed as described previously [55].

### 2.10. Morris Water Maze

Male C57BL/6J mice (*n* = 8/group) from Taconic, Lille Skensved, Denmark, 7–8 weeks of age at arrival, were used in experiments that were predominantly performed as described previously [56]. Vehicle or ACD855 (3 mg/kg; formulated in 20% DMSO in 0.1 M sodium phosphate buffer pH 8.0) was administered daily for the first four days (Day 1–4) through subcutaneous (s.c.) injections (10 mL/kg body weight). During the behavioral training days (Day 5–10), ACD855 or vehicle was administered s.c. 60 min prior to training. Moreover, during the behavioral training days, scopolamine (0.3 mg/kg) or vehicle was administered s.c. 30 min prior to training.

### 2.11. Passive Avoidance

The passive avoidance (PA) task is an associate learning paradigm that is based on Pavlovian fear-conditioning and instrumental conditioning and conducted as previously described [57] in male C57BL/6J mice (*n* = 7–8/group) from Charles River Laboratories, Sulzfeld, Germany. Vehicle or ACD855 (1, 3 or 10 mg/kg), formulated in 20% DMSO in 0.1 M sodium phosphate buffer pH 8.0, was administered daily (Days 1–4) through s.c. injections (4 mL/kg body weight). ACD856 (0.1, 0.3, 1 or 3 mg/kg) was administered by a single s.c. dose on the training day (Day 4). ACD855, ACD856, ANA-12 (0.5 mg/kg, from Sigma Aldrich, Stockholm, Sweden), physostigmine (0.025 mg/kg, from Sigma Aldrich, Stockholm, Sweden), or vehicle was administered 60 min prior to training, except for when addressing the effects on consolidation, in which case, ACD856 was dosed 5 min after the training session on day 4. Moreover, on the training day (Day 4), scopolamine or the NMDA-antagonist MK-801 (both from Sigma Aldrich, Stockholm, Sweden) at 0.3 mg/kg or vehicle was administered once s.c. 30 min prior to training. No compound was administered on the test day (Day 5), except for when addressing effects on retrieval, in which case, ACD856 was dosed 30 min prior to testing on day 5. Retention latencies and time spent in the bright compartment were determined 24 h after training (Day 5).

### 2.12. Novel Object Recognition

The novel object recognition model was conducted by Suven Life Sciences Ltd. (Hyderabad, India) using male Wistar rats (*n* = 12/group). This study was performed in compliance with the requirements and in-house animal care and usage policy of the Institutional Animal Ethics Committee (IAEC). The experiment was conducted over a 5-day period. For the first 3 days, ACD855 or vehicle were administered to the animals. On Day 4, the rats were acclimatized to their respective arenas for 20 min and then returned to their home cages for the administration of ACD855 or vehicle (1 h post habituation phase). On Day 5, the rats were administered vehicle or ACD855 1 h prior to trial, donepezil (1 mg/kg) was injected 30 min prior to trial, and scopolamine (1 mg/kg) was injected 20 min prior to trial. 20 min after scopolamine administration, the rats were subjected to the familiarization phase. The experiment was performed as described by Lueptow [58]. The discriminative index for the drug-treated groups was compared to that of the vehicle group by use of the Kruskal–Wallis test. The discriminative index is the ratio of time spent exploring the novel object divided by the sum of the time spent exploring the novel object and a familiar object in the choice trial.

### 2.13. Statistical Analysis

The overall treatment effects were analyzed using a one-way analysis of variance (one-way ANOVA) for all experiments with more than two groups, except for the Morris water maze, which was analyzed using a two-way ANOVA with repeated measurements. If significant, Tukey’s multiple comparison test was performed to assess the statistical difference between groups. An unpaired t-test was used to compare the two groups. The level of significance was set at 0.05. All studies were designed as a between subjects (independent groups) experiment (i.e., each animal was used only once). All statistics, EC_50_ or IC_50_ curves, and corresponding graphs were calculated using GraphPad Prism (GraphPad Software, Inc., La Jolla, CA, USA).

## 3. Results

### 3.1. Screening and in Vitro Characterization

To identify small molecules able to activate the TrkA receptor, a screening of approximately 25,000 compounds was performed. The mean Z’-factor for all of the screened plates was 0.36. The EC_50_ value for NGF in this assay was 1.84 ± 0.43 ng/mL (mean ± SEM, *n* = 3) in the presence of 0.1% DMSO. We obtained a total of 756 hits from the screening. The hits were further characterized in the TrkA cell assay using three different concentrations of compound (3, 10 or 30 μM) at two different concentrations of NGF (1 or 10 ng/mL). Supplemental Figure S1 shows the results from the three-point hit validation for one screening hit (Toltrazuril) from the Prestwick library. A set of 49 compounds was selected from the three-point hit validation and used to generate EC_50_-curves. Representative EC_50_-curves for six different hits at 10 ng NGF/mL are shown in Supplemental Figure S2.

**Figure 1:**
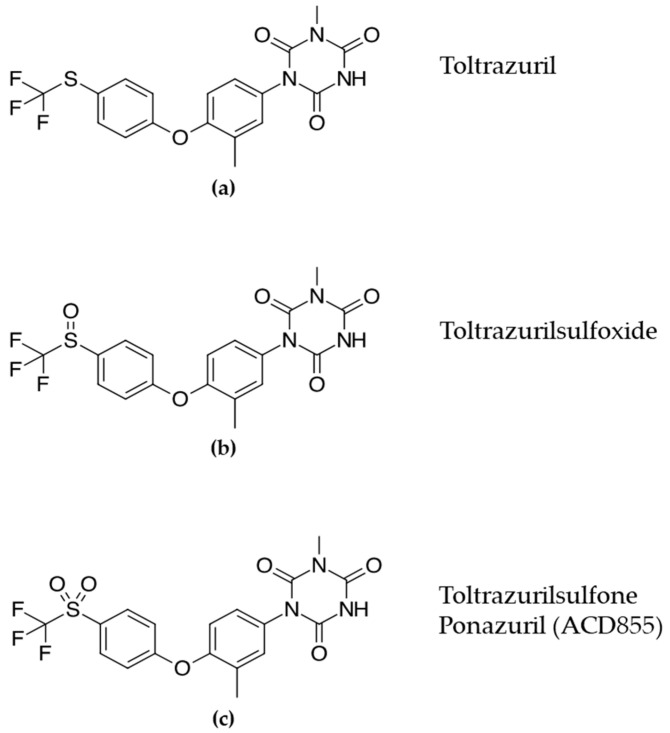
Chemical structure of (**a**) Toltrazuril and its oxidized analogues (**b**) Toltrazurilsulfoxide and (**c**) Toltrazurilsulfone.

Based upon the hit characterization and EC_50_-values, we were able to identify five distinct chemical series displaying positive modulation with low or no agonistic properties for hit validation. One of the five identified chemical series contained known triazinetriones, including the veterinary drug Toltrazuril (BAY-i 9142, Baycox^®^) and the oxidized analogues Toltrazuril-sulfoxide and Toltrazuril-sulfone (Ponazuril, BAY-i 9143, Marquis^®^), originally developed by Bayer (Figure 1). 

**Figure 2:**
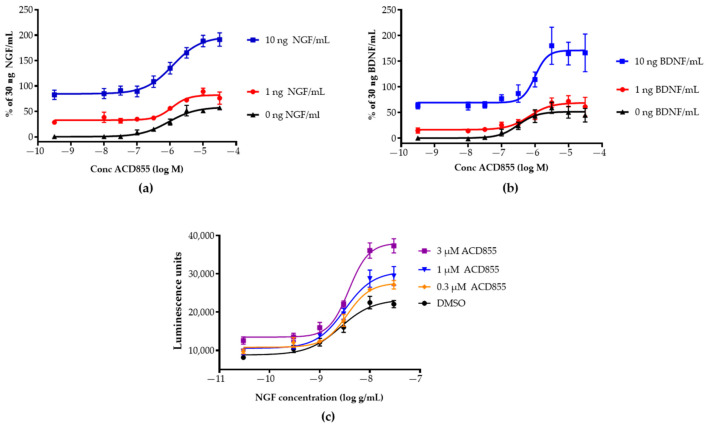
In vitro effects of ACD855 on NGF- or BDNF-signaling. U2OS cells expressing TrkA or TrkB were used to determine the effects of ACD855 at different concentrations of NGF or BDNF or to determine the effects of ACD855 on the dose–response curve of NGF. U2OS-TrkA (**a**) or U2OS-TrkB (**b**) cells (5000 cells per well) were incubated with an increasing amount of ACD855 and a fixed concentration of NGF (**a**) or BDNF (**b**), as indicated (0 ng/mL (black lines and triangles), 1 ng/mL (red lines and circles), or 10 ng/mL (blue lines and squares). 100% was defined as the activity obtained with 30 ng NGF/mL. (**c**) U2OS-TrkA cells were incubated with DMSO or with 0.3, 1, or 3 µM of ACD855, as indicated in the absence or presence of increasing amounts of NGF to achieve complete dose–response curves of NGF. Luminescence units were the total amount of luminescence obtained at all wavelengths from each well. The amount of luminescence was a direct measurement of the amount of SHC-1 interacting with the phosphorylated tyrosine residue 490 of the TrkA and thus an indirect measurement of receptor activation.

Both Toltrazuril and Ponazuril are registered veterinary products prescribed for parasitic infections in animals [59,60,61,62,63]. Ponazuril was chosen as the lead candidate for repurposing [64] based upon its pharmacokinetic properties [65], the lack of further oxidation on the sulfur-linker, convincing existing safety pharmacology and toxicology data [66], a promising pharmacological modulation of TrkA and TrkB-signaling, and it was renamed to the internal name ACD855. Interestingly, Diclazuril, another triazine-containing compound, was not active in the TrkA or TrkB assays (Supplemental Figure S3). ACD855 could effectively potentiate the response of NGF or BDNF in TrkA- or TrkB-overexpressing cell lines, respectively (Figure 2a,b). The cellular response to NGF or BDNF was measured using PathHunter^®^ assays employing β-gal enzyme fragment complementation technology. The activation of Trk-receptors leads to an intracellular interaction between the Trk-receptor and SHC-1 and thereby to the complementation of β-gal, which will render in a functional enzyme. β-gal will hydrolyze a substrate to generate luminescence, which is proportional to the number of activated receptors. Figure 2a,b demonstrates that ACD855 by itself could activate both TrkA- and TrkB-receptors to 45% without ligand when using U2OS cells overexpressing TrkA or TrkB. Moreover, using neurotrophin concentrations (10 ng of NGF or BDNF /mL) that produced almost full activation, ACD855 could activate both pathways even further with an EC_50_ of 1.9 ± 0.4 or 3.2 ± 1.2 µM (mean ± SEM, *n* ≥ 5) for TrkA (Figure 2a) or TrkB (Figure 2b), respectively. Activation of TrkA or TrkB with 30 ng/mL of ligand was set as 100% activation since no further increase in receptor activity was observed beyond 30 ng/mL. Interestingly, ACD855 could potentiate the activation of Trk-receptors beyond 100%, suggesting that there are mechanisms to further increase the kinase activity of the Trk-receptors. We have previously reported that the activation of TrkA beyond 100% is possible when using PathHunter^®^ assays. For instance, a double mutant of NGF, NGF-K95A/Q96A, could activate the TrkA receptor to a higher degree than NGF itself [55]. We could also demonstrate that ACD855 positively modulated TrkA activity when tested at three different concentrations (0.3, 1 and 3 µM), together with a wide range of NGF concentrations, as judged by Figure 2c, depicting the NGF dose–response curves in the absence or presence of increasing concentrations of ACD855.

In parallel with the preclinical development of ACD855, an ambitious lead optimization program was initiated using a screening cascade, which is outlined in Supplemental Figure S4. The screening cascade involved multiple iterations of synthesizing and testing novel triazinetrione-based compounds in the PathHunter^®^ cell-based TrkA and TrkB assays as well as in different ADME assays, including but not limited to assays for microsomal stability, hepatocyte metabolism, Caco-2 permeability, plasma protein binding, solubility as well as testing for pharmacokinetic properties and efficacy in vivo. The lead optimization program led to the discovery of several novel, potent, and in vivo active triazinetriones, including one additional candidate drug, ACD856, with an EC_50_-value of 382 ± 28 or 295 ± 35 nM (mean ± SEM, *n* ≥ 40) for TrkA or TrkB, respectively. (Table 1) [67,68,69,70]. The potency and efficacy towards other receptor tyrosine kinases (RTKs) were investigated using PathHunter^®^ cell-based assays for TrkC, FGFR1, or IGF1R. Table 1 demonstrates that the potency of ACD855 or ACD856 towards TrkC, FGFR1, and IGF1R was similar, but the efficacy on FGFR1 and IGF1R was substantially less, suggesting a selectivity towards neurotrophin receptors with respect to efficacy.

### 3.2. Mechanism of Action Studies

**Figure 3:**
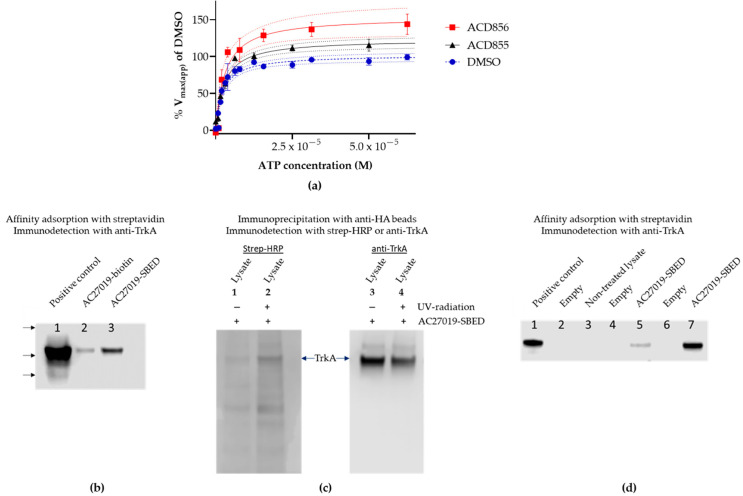
Mechanism of action of triazinetriones on TrkA. (**a**) Full-length TrkA with a HA-tag (TrkA-HA) fused to the *C*-terminus was purified through immunoprecipitation using anti-HA agarose beads. Purified TrkA-HA was incubated with DMSO (blue circles and hatched line), ACD855 (5 µM) (black triangles and solid line), or ACD856 (1 µM) (red squares and solid line) for approx. 5 min. Thereafter, ATP was added to yield the indicated concentration. Each data point is the mean ± SEM (*n* = 3). The solid lines are the curve-fit using the Michaelis–Menten equation used to calculate the apparent Vmax(app) and km(app), and the dotted lines are the 95% confidence band for each curve fit. (**b**–**d**) Affinity labeling and streptavidin adsorption of Trka. (**b**) Western blot of streptavidin adsorbed TrkA-HA non-covalent labeled with NHS-biotinylated triazinetrione compound (lane 2) or covalent labeled by UV-crosslinking of 100 µM sulfo-SBED biotinylated compound (lane 3). Detection of immunoreactive band was performed with anti-TrkA antibody. Supernatant loaded to the left (lane 1) was used as positive control for the Western blot. Arrows on the left indicate the migration of molecular weight markers corresponding to 198, 98, and 62 kDa. (**c**) Anti-HA agarose immunoprecipitation of cross-linked or non-crosslinked sulfo-SBED compound (AC27019-SBED) from cell lysate incubated with 100 µM AC27019-SBED. Lane 1, non-UV-crosslinking, and lane 2, UV-crosslinking of sulfo-SBED labeled TrkA-HA, both detected with streptavidin-HRP. Lanes 3 and 4 are loading controls of lanes 1 and 2, respectively, were TrkA-HA was detected by immunoblotting using the anti-TrkA antibody. Both blots were part of the same gel, but the membrane was cut in two pieces, and the proteins were detected by streptavidin-HRP (left panel) or by an anti-TrkA antibody (right panel). (**d**) Streptavidin adsorption of biotinylated compound bound to TrkA-HA. Lane 1, cell lysate used for positive control of immunodetection; lane 3, cell lysate without biotinylated compound was adsorbed to streptavidin-agarose as negative control; lanes 5 and 7, cell lysates containing sulfo-SBED compound UV-crosslinked to TrkA (from two different experiments) were adsorbed to streptavidin-agarose and immunoblotted using anti-TrkA antibody. Lanes 2, 4, and 6 are empty lanes to avoid cross-contamination between lanes.

The results from the cell-based assay suggested that triazinetriones could function as positive allosteric modulators of the Trk-receptors. To investigate the effect of triazinetriones on the kinase activity of the full-length receptor, hemagglutinin (HA) tagged TrkA was immunoprecipitated using anti-HA agarose beads from the lysates of SH-SY5Y cells overexpressing TrkA fused to a C-terminal HA-tag. The kinase activity in the presence of a fixed concentration of compound and increasing concentrations of ATP were used to calculate the apparent maximum rate of reaction (Vmax(app)) and the apparent Michaelis constant (Km(app)) using the Michaelis–Menten equation. As outlined in Figure 3a, ACD855 (5 µM) or ACD856 (1 µM) could significantly increase the Vmax(app) of TrkA, suggesting that the compounds act to increase the number of catalytic cycles per timepoint. The 95% confidence interval of the curve fit in Figure 3a in combination with the fact that the statistical analysis of the non-linear regression analysis rejected the null hypothesis (*p* < 0.001), suggest that the observed differences between the curves are significant. Hence, the turnover number (kcat) of the kinase reaction is increased by the binding of the triazinetriones to TrkA. Interestingly, when using only the intracellular kinase domain (ICD) composed of amino acid residues 436–790 of TrkA, we could not detect any positive modulation of the compounds on the kinase activity when using LANCE TR-FRET technology to determine kinase activity (Supplemental Figure S5). The fact that triazinetriones increased the TrkA Vmax(app), compared to DMSO-treated samples, could be an explanation for the observation that cellular Trk-activity increased above 100% after incubation with ACD855 or ACD856 (Figure 2a,b).

To further support the finding that triazinetriones interact with Trk-receptors, we performed affinity labeling of full length TrkA with two different forms of biotinylated compound. Either non-covalent interaction or UV-induced covalent crosslinking of the biotinylated probe compound to HA-tagged TrkA were used. We could demonstrate an interaction between the biotinylated compound and TrkA after either adsorption using streptavidin agarose beads or by immunoprecipitation using anti-HA agarose beads followed by Western blot detection using either anti-TrkA (Figure 3b,d) or streptavidin-HRP (Figure 3c, lane 1 and 2). As can be seen from Figure 3c (left panel), UV-crosslinking of the cell lysate incubated with the biotinylated and photoactivatable AC27019-SBED led to a more intense staining after detection with streptavidin-HRP. This demonstrates that the small molecule was indeed close enough to the Trk-receptor to allow for crosslinking. Additionally, samples from two different experiments clearly show that TrkA with AC27019-SBED bound to it could be purified by affinity adsorption using streptavidin beads and immunodetected through the use of an anti-TrkA antibody (Figure 3d, lanes 5 and 6). Control incubations with streptavidin agarose beads without the biotinylated compound did not lead to any TrkA-immunoreactive protein (Figure 3d, lane 3), confirming that the interaction between the biotinylated compound and TrkA was specific.

In order to identify the binding domain for triazinetriones on TrkA, single-site biotinylated kinase domain of TrkA was immobilized to the sensor chip of a Biacore-instrument. Subsequently, a semi-quantitative analysis of the binding of ACD856 to TrkA was performed by repeated injections with increasing concentrations (0.75–60 µM) of ACD856 onto the sensor chip. A low affinity binding site on the intracellular domain of TrkA was observed for several triazinetriones, including ACD856 with an apparent KD of 177 ± 18 µM (mean ± range, *n* = 2) (Supplemental Figure S6a). The integrity of the ATP-binding site was verified using control injections of known kinase inhibitors onto the chip (Supplemental Figure S6b). Even though the data do not support the presence of a high affinity binding site on the intracellular domain of TrkA, when biotinylated and bound to a sensor chip, the observed binding of ACD856 to TrkA suggests a low-affinity binding site. One possible explanation for the low affinity observed for ACD856 to immobilized TrkA could be that the binding site is not correctly folded, compared to the binding site on the full-length protein.

### 3.3. Facilitation of Long-Term Potentiation in Rat Hippocampal Slices

**Figure 4:**
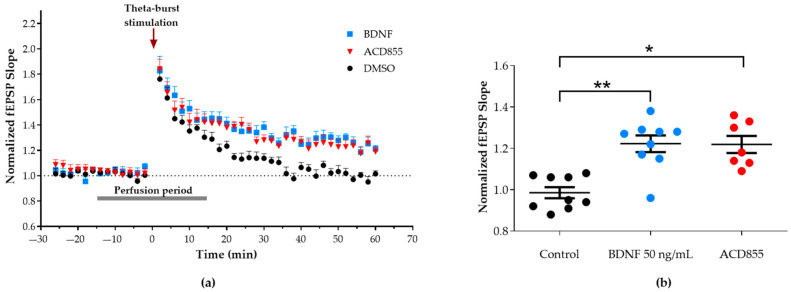
BDNF and ACD855 facilitates LTP induction in hippocampal rat slices. (**a**) Fifteen minutes after the addition of 50 ng/mL BDNF or 20 µM ACD855 to the perfusion media (aCSF), a subthreshold Ø burst stimulation (arrow) was delivered to the Schaffer collaterals. BDNF and ACD855 were washed out 15 min later, and fEPSP were monitored for 60 min after the stimulation. (**b**) Evoked synaptic responses remained potentiated for 60 min after the Ø burst stimulation in slices perfused with 50 ng/mL BDNF (blue circles, *n* = 9 slices from 7 rats) or 20 µM ACD855 (red circles; *n* = 7 slices from 5 rats) when compared to control slices (black circles, *n* = 9 slices from 9 rats). Data are expressed as mean ± SEM. * *p* < 0.05 vs. control and ** *p* < 0.01 vs. control.

It has been well-described that the transient, short-term plasticity of synaptic strength induced by a weak stimulation in hippocampal slices can turn into long-term potentiation (LTP) through the exogenous application of BDNF [71,72,73]. Therefore, we examined if a positive modulator of TrkB, such as ACD855, could facilitate LTP induction similar to BDNF using rat hippocampal slices. For this purpose, a single train of Ø burst stimulation, which induced a transient potentiation of synaptic strength and measured as an increase in the normalized fEPSP slope, was delivered at the Shaffer collaterals while recording at the stratum radiatum of CA1. In the control slices, the recorded potentiation decreased to the baseline level after 60 min. In contrast, the addition of 20 µM ACD855 15 min before Ø burst stimulation resulted in a long-lasting potentiation of the evoked synaptic responses (1.21 ± 0.03-fold increase; *n* = 7 slices from 5 rats), which was similar to that observed by addition of 50 ng/mL BDNF (1.22 ± 0.04-fold increase; *n* = 9 slices from 7 rats) shown in Figure 4. Neither 50 ng/mL BDNF or 20 µM ACD855 affected basal evoked synaptic transmission or the peak amplitude reached immediately after potentiation. 

### 3.4. Microdialysis

In order to evaluate the effects of the local administration of ACD855 on extracellular neurotransmitter levels, including acetylcholine (ACh), glutamate (Glu), dopamine (DA), noradrenaline (NA), and serotonin (5-HT), in the ventral hippocampus (vHPC) of awake rats, microdialysis was performed. Local infusion of ACD855 over a period of four hours induced a gradual but significant 40% increase in extracellular ACh levels in the vHPC of freely moving rats, compared to vehicle- (aCSF) treated animals (*p* < 0.05; unpaired two-tailed t-test, Supplemental Figure S7a). During the microdialysis experiments, it was observed that the effect of ACD855 infusion on the increase in ACh levels was markedly delayed and mostly occurred during the wash out period. This effect was found to be due to the unspecific adsorption of ACD855 on the inner walls of the tubing and the probe in the microdialysis system. Based on these results, it appeared more relevant to compare the late period between 6 and 10 h of ACD855 infusions to the control periods of 0 to 4 h. The levels of glutamate, noradrenaline, dopamine, and 5-hydroxytrypatmine were not affected (Supplemental Figure S7b,c).

### 3.5. In Vivo Behavioral Cognitive Models

**Figure 5:**
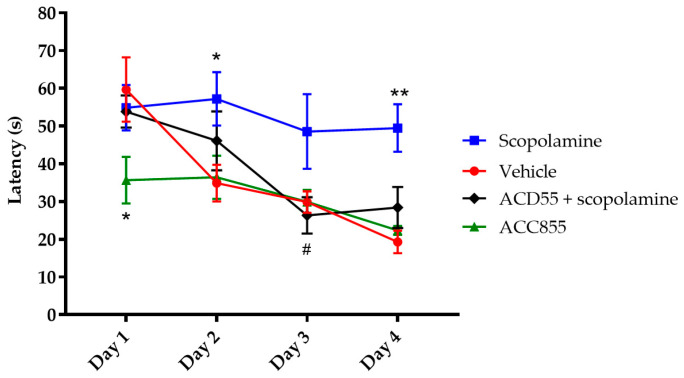
Effects of ACD855 in the Morris water maze. Subsequent to the pretraining, four days of spatial training in the water maze took place, whereby the animals received four daily trials starting at four different starting points around the water tank. Time to locate the platform (latency) was measured. Vehicle-treated animals (red line and circle) learned the task rapidly, while scopolamine- (0.3 mg/kg) treated animals (blue line and squares) had a highly significant impairment in swim latency, (* *p* < 0.05 (Day 2) and ** *p* < 0.01 (Day 4); respectively) in comparison to the control group. ACD855 (3 mg/kg) completely blocked the impairing effect of scopolamine on swim latency (black line and diamonds) (# *p* < 0.05 (Day 3)). ACD855 on its own improved cognitive function compared to vehicle, as there was rapid learning exhibited on Day 1 that significantly differed from the control animals (green line and triangles) (* *p* < 0.05 (Day 1)).

Considering the positive modulatory effects of ACD855 or ACD856 on TrkB in combination with the effects on LTP and the increased levels of acetylcholine, we were interested in evaluating the effects of the compounds on cognitive function. Thus, the compounds were characterized in a panel of rodent models to assess the potential effects on cognition in vivo. Figure 5 shows the effect of ACD855 in the Morris water maze (MWM), a hippocampal-dependent test for spatial memory. ACD855 (3 mg/kg) significantly blocked the overall cognitive impairment induced by scopolamine, as measured by swim latency (Figure 5). Scopolamine is a nonspecific muscarinic antagonist with anticholinergic effects that is often used to induce amnesia in animals. Interestingly, ACD855 by itself seemed to improve cognitive function compared to vehicle, as there was rapid learning exhibited during the 4 trials on Day 1 that significantly differed from control animals. There were no significant differences in swim speed, and there was only an improvement trend in the retention test (probe trial) between the control- and treated- groups (Supplemental Figure S8a,b).

**Figure 6:**
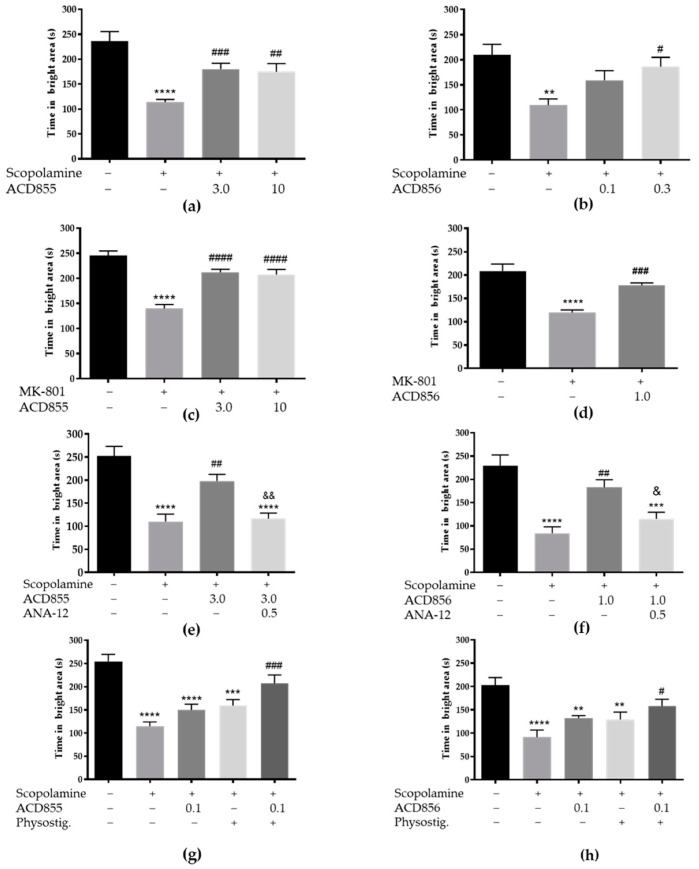
ACD855 or ACD856 reverses memory-impairment in the passive avoidance task in mice. ACD855 (3 or 10 mg/kg) was administered s.c. for 4 consecutive days, whereas ACD856 (0.1, 0.3 or 1 mg/kg) was administered as a single dose 60 min prior to the experiment in combination with scopolamine (0.3 mg/kg, s.c.), MK-801 (0.3 mg/kg, i.p.), ANA-12 (0.5 mg/kg, s.c), or physostigmine (0.0125 mg/kg, s.c). Memory was assessed by measuring the total time the animals spent in the bright compartment, the maximum being 300 s. (**a**,**b**) ACD855 or ACD856 reversed scopolamine-induced memory impairment at the indicated doses. ** *p* < 0.01 or **** *p* < 0.0001 for scopolamine vs. the control group. #### *p* < 0.0001, ## *p* < 0.01, or # *p* < 0.05 for compound and scopolamine-treated animals vs. the scopolamine-treated group. (**c**,**d**) ACD855 or ACD856 reversed MK-801 induced memory impairment at the indicated doses. **** *p* < 0.0001 for MK-801 vs. the control group. ### *p* < 0.001 or ## *p* < 0.01 for compound- and MK-801-treated animals vs. the MK-801-treated group. (**e**,**f**) ANA12 inhibited ACD855- or ACD856-induced memory improvement. Animals were treated with scopolamine, ACD855 or ACD856 or ANA-12 as indicated (mg/kg). **** *p* < 0.001 for scopolamine vs. the control group. ## *p* < 0.01 for ACD855- and scopolamine-treated animals vs. the scopolamine-treated group, & *p* < 0.05 or && *p* < 0.01 for 855 and scopolamine- treated animals vs. ANA-12-, ACD855-, and scopolamine-treated group. (**g**,**h**) Physostigmine and ACD855 or ACD856 acted in an additive manner to reverse scopolamine-induced impairment. **** *p* < 0.0001 for scopolamine vs. the control group. **** *p* < 0.0001 or ** *p* < 0.01 for scopolamine + physostigmine vs. the control group. *** *p* < 0.001 or ** *p* < 0.01 for scopolamine + ACD855 or ACD856 vs. the control group and ### *p* < 0.001 or # *p* < 0.05 for scopolamine-, ACD856-, and physostigmine-treated animals vs. the scopolamine-treated group.

Next, we used an associative learning model, the passive avoidance model, in the mice, whereby potential pro-cognitive effects of ACD855 or ACD856 were evaluated. Data from naïve young healthy animals indicated that none of the two compounds had any pro-cognitive effects in naive animals or on other measured behavioral parameters, e.g., motor activity or exploration (Supplemental Figure S9a–c). However, administration of ACD855 at a dose of 3 or 10 mg/kg once daily for four days or a single s.c. injection of ACD856 (0.3 mg/kg) were able to significantly attenuate the memory-impairing effects of scopolamine, as measured by the time spent in the bright compartment (Figure 6a,b). ACD855 or ACD856 could also ameliorate the effects of MK-801, an N-methyl-D-aspartate (NMDA) antagonist in the same behavioral model (Figure 6c,d). The effects of ACD855 or ACD856 on scopolamine-induced memory impairment were dose-dependent and blocked by ANA-12, a reported TrkB antagonist (Figure 6e,f). Furthermore, combined administration of low doses of ACD855 or ACD856 and physostigmine (0.025 mg/kg), which by themselves had no significant effect on scopolamine-induced memory impairment, were able to significantly reverse the memory impairment induced by scopolamine (Figure 6g,h). Physostigmine is an alkaloid functioning as a reversible acetylcholinesterase inhibitor, and thus it increases the levels of acetylcholine available in the synaptic cleft.

**Figure 7:**
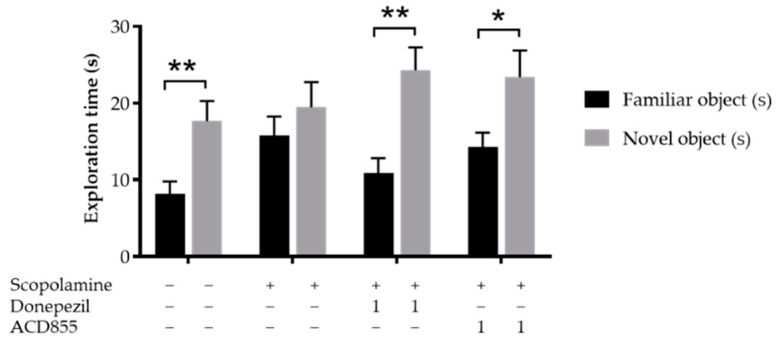
Effects of ACD855 and donepezil on object recognition in Wistar rats. ACD855 was administered for 4 consecutive days using 1 mg/kg, whereas donepezil was administered 30 min before the trial, both in combination with 1 mg/kg scopolamine. Groups treated with scopolamine and donepezil or ACD855 spent significantly greater time with the novel object. Black bars indicate familiar object and grey bars indicate novel object. ** *p* < 0.01 or * *p* < 0.05 for time spent on novel objects vs time spen on familiar objects.

In order to test the nootropic effects of ACD855 in a model of episodic memory, we used scopolamine induced memory deficit in the object recognition task using adult Wistar rats. On the test day, vehicle-treated rats spent a significantly greater time with the novel object in comparison to the familiar object. Rats treated with scopolamine did not discriminate the novel object from the familiar object, whereas animals treated with Donepezil or ACD855 (1 mg/kg) followed by scopolamine spent significantly more time with the novel object (Figure 7). Donepezil is a reversible acetylcholinesterase inhibitor, thus inhibiting the breakdown of acetylcholine.

**Figure 8:**
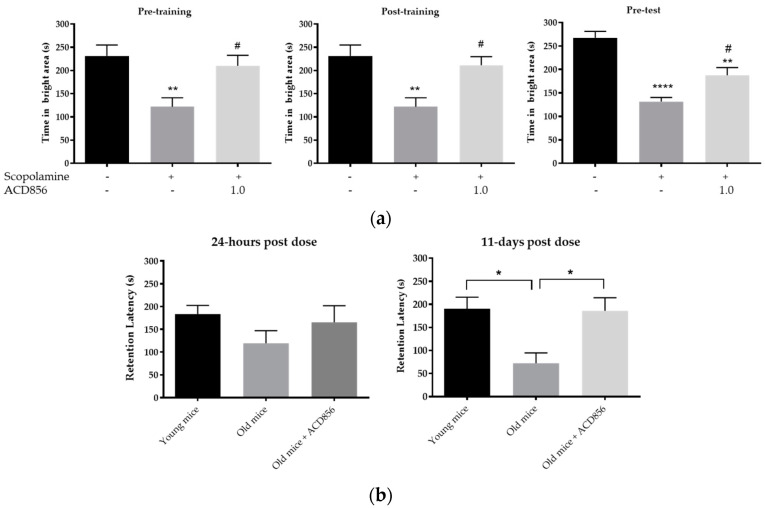
ACD856 improves the acquisition, consolidation, and retrieval of memories. (**a**) Effects of ACD856 on acquisition, consolidation, or retrieval on scopolamine-induced amnesia in the passive avoidance model. The memory was assessed by measuring the total time that the animals spent in the bright compartment, the maximum being 300 s. Vehicle or ACD856 were administered as a single dose by subcutaneous (s.c.) injection (4 mL/kg body weight) at different timepoints in relation to the training session. On the day of training (Day 1), ACD856 or vehicle was administered either 60 min prior to training (pre-training, left graph) or 5 min post-training (post-training, middle graph). When evaluating the effects on the retrieval of memories on day 2, either vehicle or ACD856 was administered 30 min before the test session (pre-test). On the day of training, scopolamine at 0.3 mg/kg, or vehicle was administered subcutaneously 30 min prior to training. ** *p* < 0.01 or **** *p* < 0.0001 for scopolamine- vs. the vehicle-treated group (black bars). # *p* < 0.05 for compound and scopolamine-treated animals vs. the scopolamine-treated group. (**b**) ACD856 reversed aged-induced memory impairment at 24-h and 11 days post-dose. Left panel, the old mice displayed lower retention latency than the young mice, but it was not statistically significant. A single injection of ACD856 at a dose of 3 mg/kg induced a slight increase in retention latency in comparison to vehicle-treated old mice. Right panel, the retention latency was significantly higher in control young mice and the ACD856-treated group with old animals in comparison to the old control group when tested 11 days after the first retention test (* *p* < 0.05).

We also addressed the different modalities of memory formation in the passive avoidance model by dosing ACD856 at different time points in relation to the training session. A single s.c. injection of ACD856 (1 mg/kg) pre- or post-training or pre-test significantly reversed the impairment of scopolamine on the time spent in the bright compartment (*p* < 0.05; Figure 8a). Administration of ACD856 (1 mg/kg) pre- or post-training, but not pre-test, also significantly reversed the impairment of scopolamine on retention latency (*p* < 0.05; Supplemental Figure S10a–c).

Finally, we addressed the pro-cognitive effects of ACD856 in C57BL/6J mice who displayed a natural age-related decline in cognition. The data in Figure 8b show that 18-month-old mice had lower retention latency than young mice (8 weeks old). A single s.c. injection of ACD856 (3 mg/kg) induced a non-significant increase in retention latency in comparison to vehicle-treated old mice (Figure 8b, left graph) when the memory test was performed 24 h after the training session. In contrast, when the memory performance was assessed 11 days after the first training session in the same animals, there was a clear and significant difference between the young animals and the old animals as well as between the ACD856-treated old animals and the vehicle-treated old animals (*p* < 0.05; Figure 8b, right graph), suggesting that ACD856 enabled the 18-month-old animals to retrieve memory to the same extent as young (2-month-old) animals.

## 4. Discussion

Currently, marketed AD therapeutics are limited to two different categories of symptomatic therapies: acetylcholinesterase inhibitors (AChEIs) and the NMDA antagonist memantines. A recent study where 11,652 Alzheimer’s dementia patients using AChEIs were compared to 5826 non-AChEI users in a 5-year follow up, demonstrated that the AChEIs-users had higher MMSE and a lower mortality rate than non-users [74]. Additional studies have shown that treatment using AChEIs is associated with reduced mortality in AD patients [75,76], a reduced risk for stroke [76], and a 37% reduction of cardiovascular events in persons with dementia treated with AChEIs [77]. On the other hand, a meta-analysis of randomized controlled trials with a mean duration of 30 weeks and a few randomized controlled trials (RCTs) lasting longer (mean 8 months) showed no significant effect of AChEI on cognition [78]. Although the results from the next generation of anti-amyloid treatments are eagerly awaited, many of the clinical trials using substances targeting the amyloidogenic pathway have hitherto failed [79], and there is an urgent need to develop novel therapeutics targeting other, non-Aβ targets. Thus, we report here on the identification of ACD855 and ACD856 as novel positive allosteric modulators of the neurotrophin receptors with effects on synaptic function and cognition in several preclinical models.

In AD, the metabolism of the NGF has been found to be altered, resulting in an accumulation of pro-NGF levels stimulating apoptosis via the p75 receptor and a reduction of mature NGF stimulating cell survival via the TrkA receptor [27]. A decreased ratio of TrkA/p75 was found in AD [80], which therefore may lead to increased apoptotic signaling induced by pro-NGF. These alterations have also been observed in AD-like mice models [81]. Moreover, in the AD11 mice model, which expresses anti-NGF antibodies specifically in the brain, the lack of mature NGF leads to early inflammation and neurodegeneration that is characteristic to Alzheimer’s disease [82].

We identified a novel mechanism for the positive allosteric modulation of Trk receptors and promising clinical candidates that potentiated the effects of NGF or BDNF in a cellular context. The compounds bind to the Trk receptors, thereby increasing the catalytic efficiency of the receptor’s kinase activity. The reason for the relative low affinity binding of the triazinetriones in the Biacore-experiments compared to the cellular EC_50_-vaules could be explained by the fact that the conformation of the binding site for triazinetriones on TrkA was not fully correct due to the lack of the juxta membrane and the transmembrane regions in the construct that was used for the Biacore studies. The lack of effects of triazinetriones on the kinase activity of TrkA-ICD but the significant effects on the full-length receptor supports results with Biacore and suggests that the binding pocket of triazinetriones on the ICD of TrkA does not have the right conformation to allow for a high affinity and functional modulatory site. Thus, studies aimed at the modulation of the function of the Trk-receptors should preferably include the use of full-length receptors.

The fact that ACD855 (Ponazuril) has been used as a safe and well tolerated veterinary medicine for long time [62,83] has made it a suitable candidate for repurposing in humans as a cognitive enhancer. The compound was able to facilitate LTP in a manner that is similar to BDNF itself and increased the levels of acetylcholine in the ventral hippocampus, suggesting that ACD855 can have an effect on both glutamatergic synaptic function as well as cholinergic function. Furthermore, the pro-cognitive effect of ACD855 and ACD856 were additive to physostigmine. This is interesting, especially when considering the fact that the local infusion of ACD855 led to increased acetylcholine levels in the hippocampus. Additionally, the observed pro-cognitive effects in vivo are dependent on TrkB since the effects could be blocked by the TrkB inhibitor ANA12. The fact that ACD855 and ACD856 could ameliorate the effects of MK-801 and scopolamine suggest that both cholinergic and glutamatergic processes are affected by the compounds. It has been suggested that an approach combining cholinergic with glutamatergic treatment could be a beneficial symptomatic treatment for Alzheimer’s disease [84]. The underlying mechanism of triazinetriones on cognition seems to involve both cholinergic activities, presumably mediated via NGF/TrkA, and an effect on synaptic transmission, presumably mediated via BDNF/TrkB. The significant effects observed in multiple cognitive domains including spatial, associative, and episodic memory as well as the effects on learning, consolidation, and retrieval mechanisms would also indicate an effect mediated through multiple mechanisms and at different anatomical sites in the brain.

The effects of the interaction of ACD855 or ACD856 with the receptor, leading to increased TrkA activity, translated well into increased cellular neurotrophic signaling and cognitive effects in vivo, as shown in multiple rodent cognition models. Furthermore, Neither ACD855 or ACD856 show any effect on cognitive function or general behavior in naive animals when using the passive avoidance model. Thus, the pharmacological effect of the compounds is probably only manifested in animals that have impaired memory function, exemplified by scopolamine- or age-induced amnesia, or in situations when there are increased levels of neurotrophins binding to the Trk-receptors, such as in the Morris water maze.

The difference between the lack of effects of ACD855 in naïve animals using the passive avoidance models compared to the positive effect of ACD855 on spatial memory in naïve animals using the Morris water maze model could perhaps be explained by a potential effect of ACD855 during the pretraining session in the water maze. Pre-training is commonly conducted to facilitate procedural learning in this spatial navigation task. Considering that ACD855 was given during the pre-training sessions, it is possible that the compound could have significantly affected the rate of acquisition from the start by having a positive effect on procedural learning. The latency data indicate a very rapid onset of effect in this treatment group at least. Another possibility could relate to the difference in complexity of the two models. Although both models are dependent on a functional hippocampal circuitry, it was previously shown that the conditional knockout of BDNF in the hippocampus and anterior forebrain in animals did not affect their capability of performing less complex learning test involving aversive stimuli. However, the animals displayed impaired learning in more complex or stressful models such as water maze [85], suggesting that some complex tasks are dependent on BDNF/TrkB-signaling in the hippocampus to a larger extent than less complex tasks. For instance, one study demonstrated a rapid and transient increase in BDNF mRNA in the CA1 region of the dorsal hippocampus after inhibitory avoidance training but failed to show increased levels of BDNF protein [86]. Nevertheless, anti-BDNF antibodies infused into the same area prior to training but not immediately or after training, demonstrated amnesic effects [86]. In contrast to avoidance training, the BDNF protein and the phosphorylation of TrkB was enhanced in the dentate gyrus part of the hippocampus after spatial training using the Morris water maze [87]. Finally, it was demonstrated early on that BDNF plays an important role in the formation of spatial memory since BDNF knockout animals display severe memory impairment using the Morris water maze [88]. Thus, the effects of ACD855 on spatial learning in the water maze model in naïve animals could perhaps be explained by a higher degree of BDNF/TrkB signaling in this complex spatial navigation task than in naïve animals in less complex models. 

The results from our preclinical studies together with the existing safety and toxicology data demonstrated the clear potential of ACD855 and ACD856 to improve cognition. Considering the broad role of neurotrophins, including supportive and neuroprotective activity, triazinetrione-based positive allosteric modulators of Trk receptors may also have an additional upside of achieving disease-modifying effects in neurodegenerative disorders like AD or PD. 

In summary, we have identified a novel mechanism for triazinetrione derivates leading to the increased activity of the neurotrophin receptors and identified ACD855 and ACD856 as cognitive enhancers in various animal models through the enhancement of Trk-signaling. ACD856 is currently in a clinical phase 1 study, which will be reported elsewhere.

## 5. Patents

See references [64,67,68,69,70].

## Figures and Tables

**Table 1:** Summary of EC_50_ values (µM) and the efficacy (%) of ACD855 or ACD856 on the activation of neurotrophin (TrkA, TrkB or TrkC), insulin-like growth factor 1 receptor (IGF1R), and fibroblast growth factor receptor (FGFR1).

	TrkA		TrkB		TrkC		IGF1R		FGFR1	
	EC_50_	Effect	EC_50_	Effect	EC_50_	Effect	EC_50_	Effect	EC_50_	Effect
ACD855	1.9	144%	3.2	319%	0.8	56%	0.9	25%	0.8	44%
ACD856	0.38	185%	0.29	176%	0.33	328%	0.25	104%	0.19	117%

## Data Availability

The data presented in this study are available on request from the corresponding author. The data are not publicly available due to company confidentiality reasons.

## Supplementary Materials

The following are available online at https://www.mdpi.com/article/10.3390/cells10081871/s1, Figure S1: Effects of Toltrazuril on U2OS-TrkA/p75 cells, Figure S2: Representative effects of selected screening hits, Figure S3: Schematic illustration of the key activities in the hit identification and validation process as well as key steps in the lead optimization, Figure S4: Effects of diclazuril on U2OS-TrkA/p75 cells activated with 10 ng NGF/mL, Figure S5: Effects of Toltrazuril on the kinase activity of the intracellular domain of TrkB, Figure S6: Effects of ACD856 on binding to single site biotinylated TrkA immobilized to the sensor chip of a Biacore instrument., Figure S7: Effects of ACD855 on neurotransmitters in the ventral hippocampus as judged by microdialysis., Figure S8: Effects of ACD855 in the Morris water maze, Figure S9: Effects of ACD855 on latency activity or exploration in naïve animals using the passive avoidance task, Figure S10: Effects of ACD856 on acquisition, consolidation or retrieval on scopolamine-induced amnesia in the passive avoidance model as measured by retention latency.
