# Supplementary material for: Identification of Novel Positive Allosteric Modulators of Neurotrophin Receptors for the Treatment of Cognitive Dysfunction
Source: Cells. 2021 Jul 23;10(8):1871. doi: 10.3390/cells10081871 (PMC8391421; doi:10.3390/cells10081871)
Supplement: Supplementary file 1 [file cells-10-01871-s001.zip › cells-1289881-supplementary.pdf]

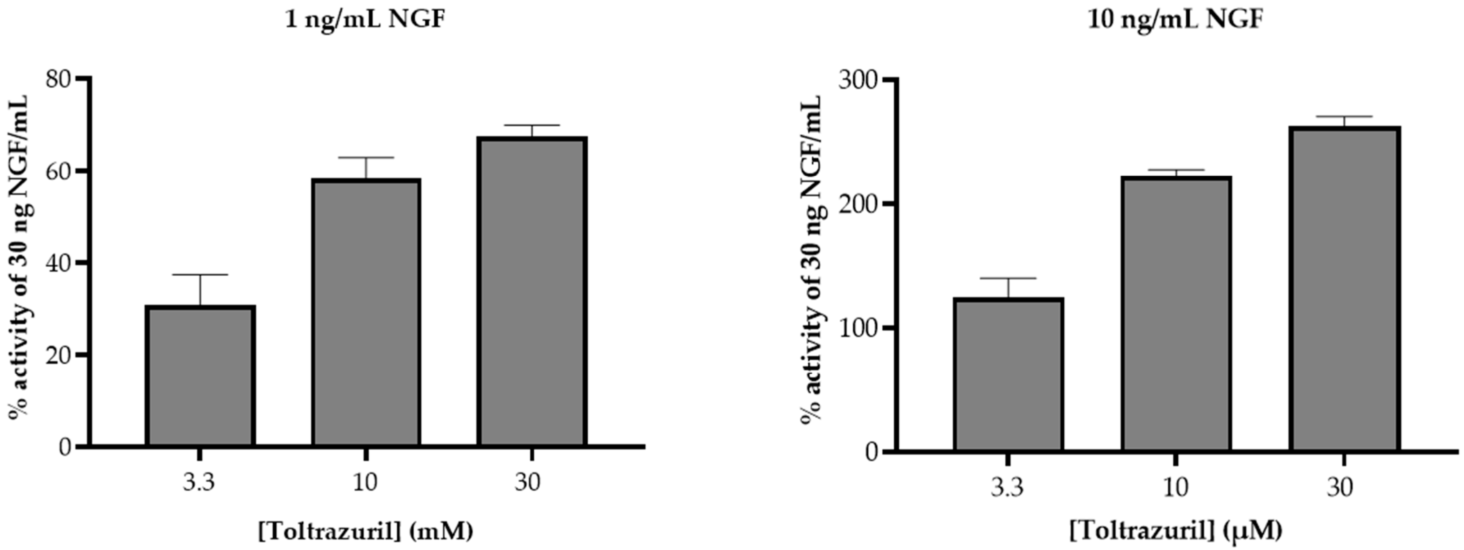

Supplemental figure S1. Effects of Toltrazuril on U2OS-TrkA/p75 cells activated with 1 **(a)** or 10 **(b)** ng NGF/mL. The positive effects of Toltrazuril U2OS-TrkA/p75 cells were examined by a three-point dose-response at two different concentrations of NGF, 1 or 10 ng NGF/mL using 10,000 cells and an incubation time of 3 h. 100% was defined as the activity obtained with 30 ng NGF/mL. The data show that Toltrazuril was able to increase the activity of TrkA at both 1 and 10 ng/mL of NGF.

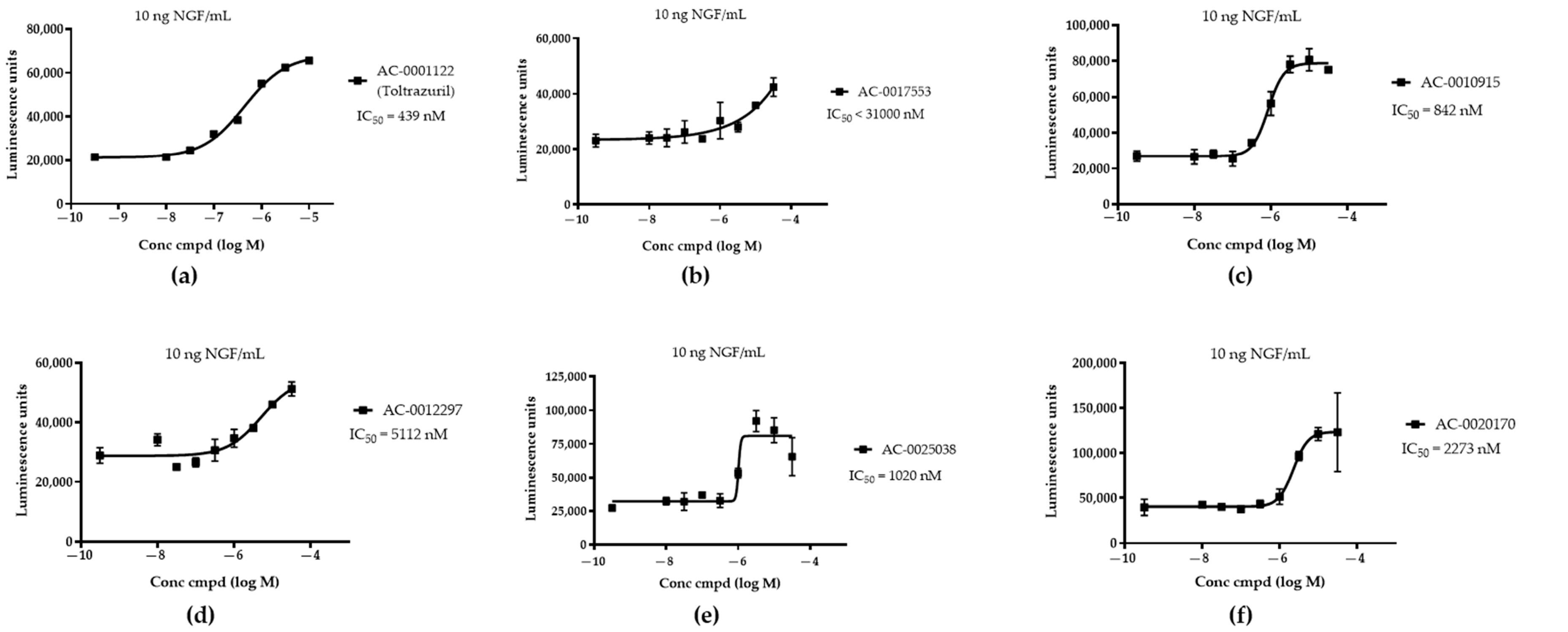

Supplemental figure S2. Representative effects of selected screening hits **(a-f)** on U2OS-TrkA/p75 cells activated with 10 ng NGF/mL. Effects of screening compounds on U2OS-TrkA/p75 cells were examined by dose-response curves of compounds using 10,000 cells and an incubation time of 3 hours. The non-linear regression curve was fitted to the data point and used to calculate the displayed EC_{50} values.

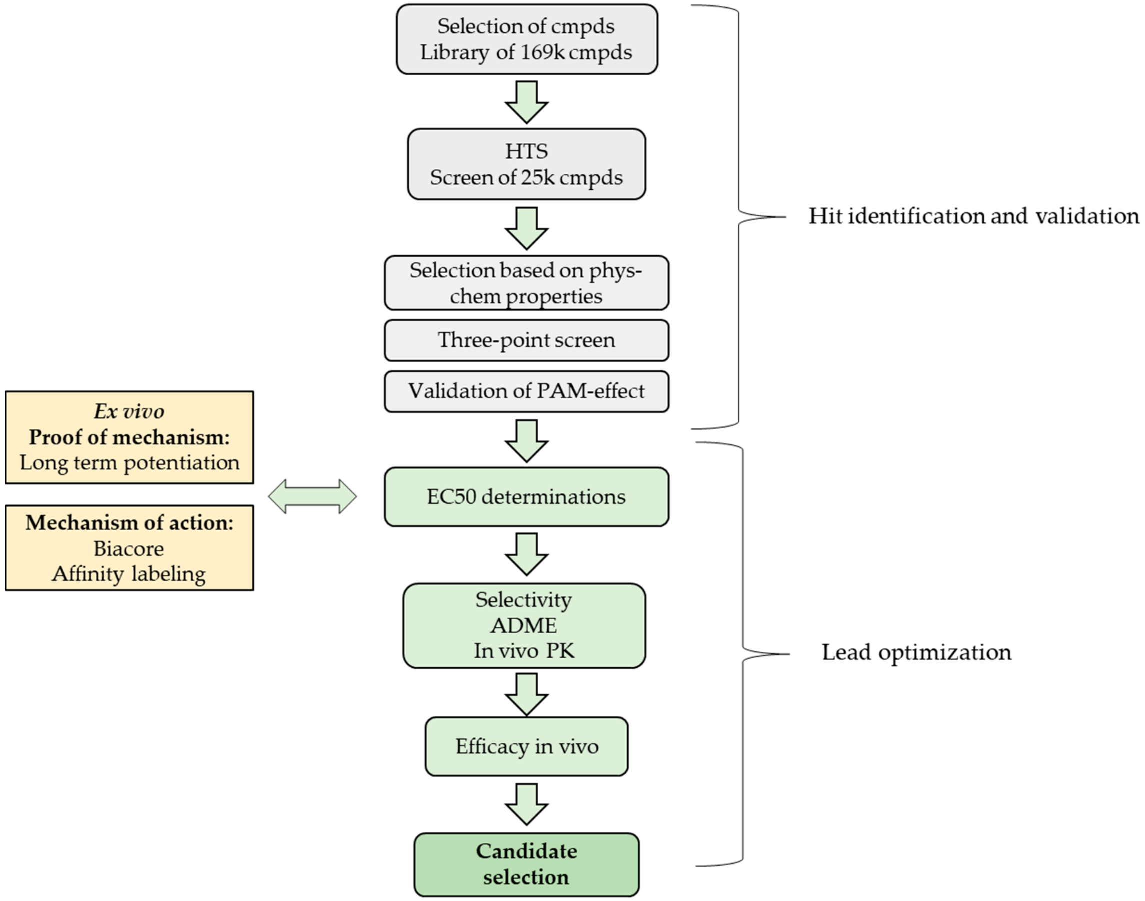

Supplemental figure S3. Schematic illustration of the key activities in the hit identification and validation process as well as key steps in the lead optimization.

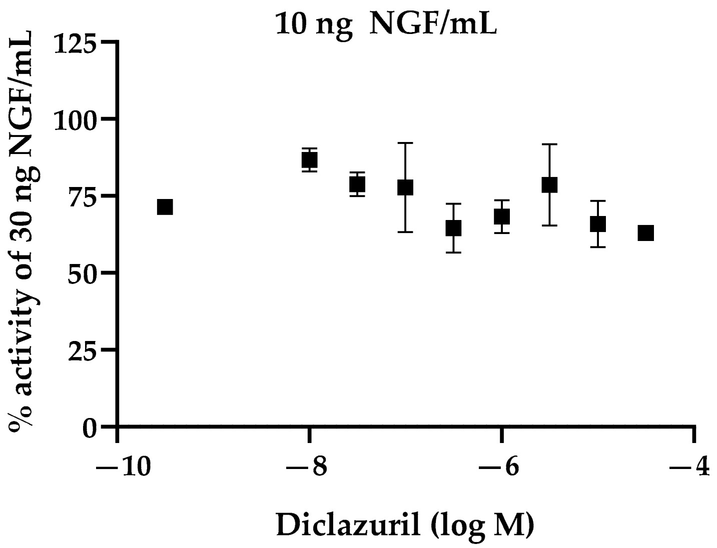

Supplemental figure S4. Effects of diclazuril on U2OS-TrkA/p75 cells activated with 10 ng NGF/mL. Effects of Diclazuril on U2OS-TrkA/p75 cells were examined by a dose-response curve using 10,000 cells and an incubation time of 3 h. The non-linear regression curve could not be fitted to the data points, suggesting a lack of effect of the compound on cellular NGF-induced TrkA activity.

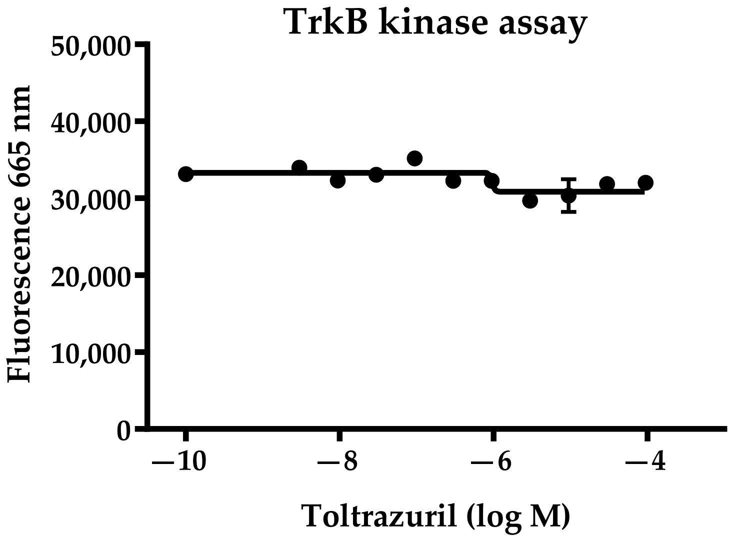

Supplemental figure S5. Effects of Toltrazuril on the kinase activity of the intracellular domain of TrkB. The biochemical TrkB-kinase assay was performed using 10 different concentrations of Toltrazuril. The intracellular kinase domain of TrkA was supplied by Carna Biosciences and the artificial peptide substrate ULight™-TK peptide substrate and the anti-phosphotyrosine (PT66) antibody were provided by PerkinElmer. TrkB kinase reaction was run for 90 min at room temperature before addition of a 5 µL stop solution containing EDTA and detection. Samples were incubated for 60 min at room temperature before fluorescence was measured using an with an excitation wavelength of 337 nm and an emission wavelength of 665 nm. The non-linear regression curve-fit is displayed.

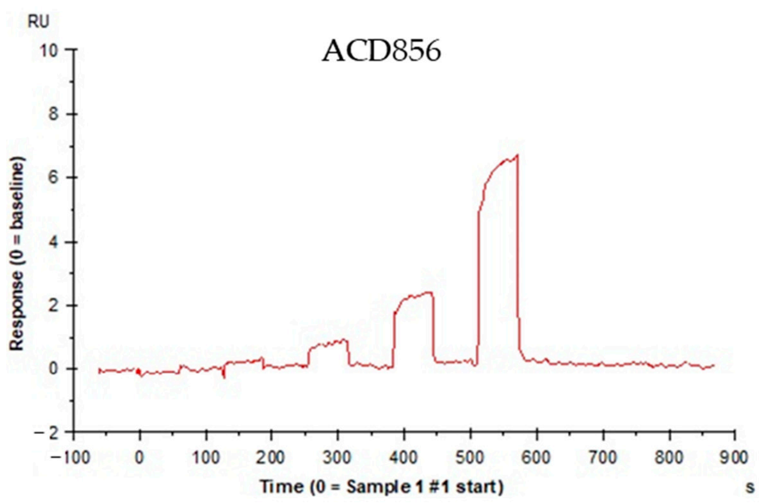

(a)

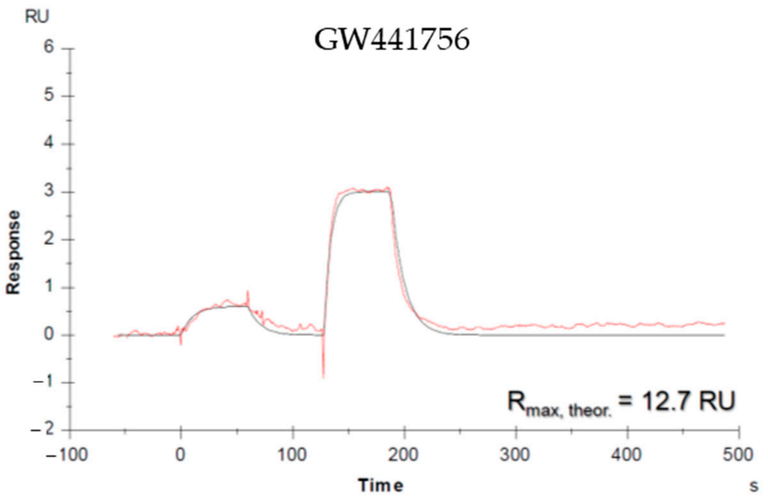

(b)

Supplemental figure S6. Effects of ACD856 on binding to single site biotinylated TrkA immobilized to the sensor chip of a Biacore instrument. **(a)** A semi-quantitative analysis of the binding of ACD856 to immobilized TrkA was performed by repeated injections with increasing concentrations (0.75–60 µM) of ACD856 onto the sensor chip. The sensorgram is one out two independent experiments. The binding of ACD856 to TrkA was approximately 30% of the calculated theoretical Rmax value and extrapolation of the responses yielded an apparent KD of 177 ± 18 µM. **(b)** The integrity of the ATP-binding site was verified by control injections of two concentrations of a known ATP-competitive kinase inhibitor (GW441756) onto the chip. The results demonstrate good integrity of the ATP-binding site of immobilized TrkA.

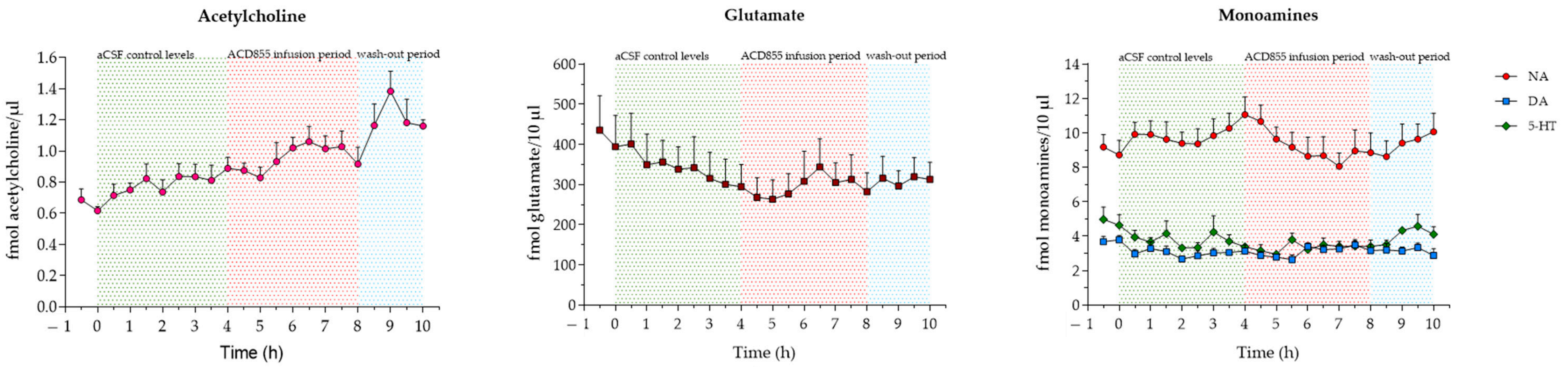

(a) (b) (c)

Supplemental figure S7. Effects of ACD855 on neurotransmitters in the ventral hippocampus as judged by microdialysis. Effect of local infusion of 30 µM ACD855 in aCSF through the microdialysis probe during the 4-hours period (4–8 h, marked as a red-dotted area) and the following 2-hours wash-out period (marked as a blue-dotted area) compared to the control, 0–4 h period (marked as a green-dotted area) on extracellular levels of **(a)** Acetylcholine, **(b)** glutamate or **(c)** noradrenaline/norepinephrine (NA), dopamine (DA) or 5-hydroxytrypatmine (5-HT) in the ventral hippocampus of awake rats measured as actual concentrations found in the microdialysates. There was a increase of the levels of acetylcholine, especially between the timepoints 6 to 10 hours. There were no effects on glutamate or the monoamines, NA, DA or 5-HT.

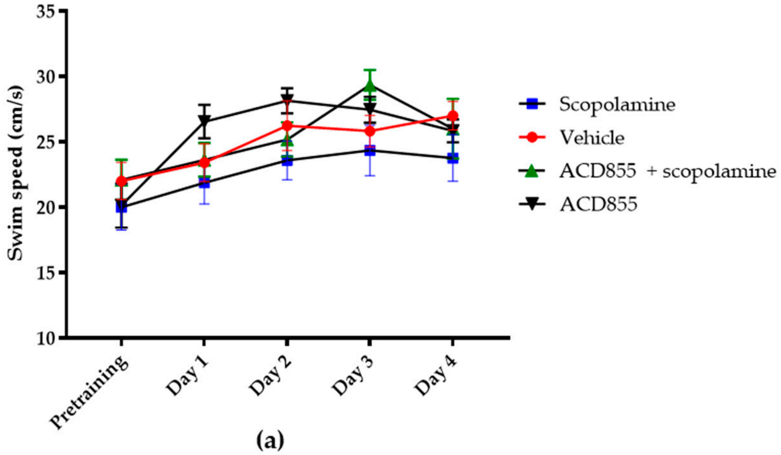

(a)

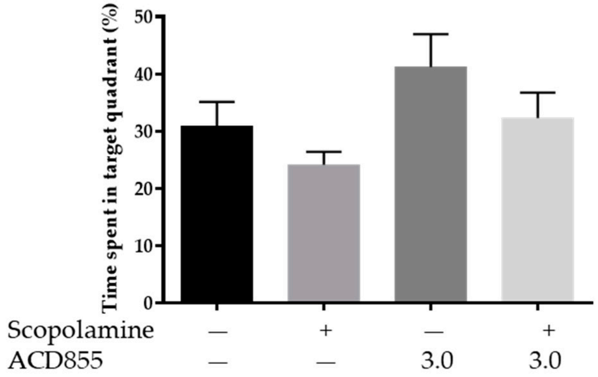

(b)

Supplemental figure S8. Effects of ACD855 in the Morris water maze. **(a)** Subsequent to the pretraining, four days of spatial training in the water maze took place whereby the animals received four daily trials starting at four different starting points around the water tank. Swim speed was recorded. There were no significant differences in swim speed between the different groups. Vehicle treated animals (red line and circle), scopolamine treated animals (0.3 mg/kg), ACD855 treated 3 mg/kg(black line and diamonds), ACD855 and scopolamine treated animals (green line and triangles). **(b)** Effects of scopolamine and ACD855 on probe trial Day 5. There were no significant differences between the treatment groups in the probe trial.

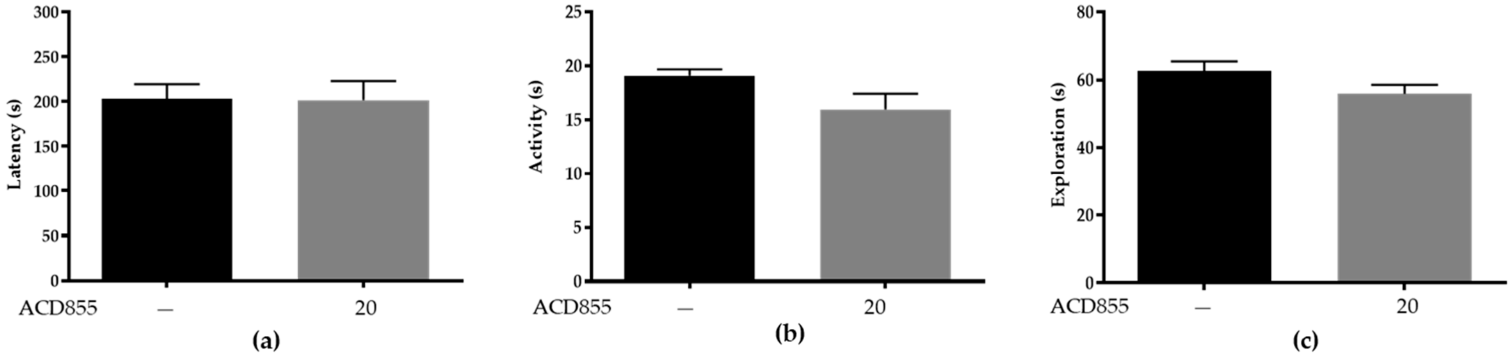

Supplemental figure S9**.** Effects of ACD855 on latency activity or exploration in naïve animals using the passive avoidance task. ACD855 (20 mg/kg) was administered as a single dose in naïve animals 60 min prior to the passive avoidance experiment. The memory was assessed by measuring the retention latency **(a),** the motor activities was assessed by measuring the activity **(b)** or exploration **(c)** .

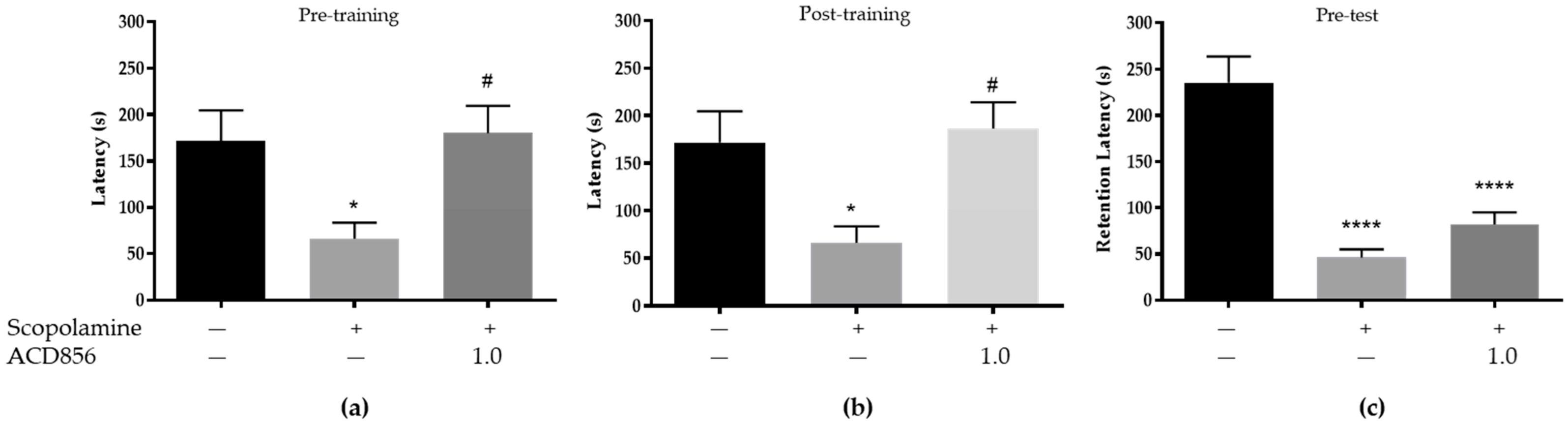

Supplemental figure S10**.** Effects of ACD856 on acquisition, consolidation or retrieval on scopolamine-induced amnesia in the passive avoidance model as measured by retention latency. Vehicle or ACD856 were administered as a single dose by subcutaneous (s.c.) injection (4 mL/kg body weight) at different timepoints in relation to the training session. On the day of training (day 1), ACD856 or vehicle was administered either 60 min prior to training (pre-training) **(a)** or 5 min post-training (post-training)**(b)**. When evaluating effects on retrieval of memory on day 2, either vehicle or ACD856 was administered 30 min before test session (pre-test)**(c).** On the day of training, scopolamine at 0.3 mg/kg, or vehicle, was administered subcutaneously 30 min prior to training. * $p < 0.05$ for scopolamine vs the vehicle treated group (black bars). **** $p < 0.001$ for scopolamine vs the vehicle treated group (black bars). # $p < 0.05$ for compound and scopolamine treated animals vs the scopolamine treated group.
